# Expression of Concern on ‘DICER-dependent biogenesis of let-7 miRNAs affects human cell response to DNA damage via targeting p21/p27’

**DOI:** 10.1093/nar/gkac553

**Published:** 2022-06-18

*Nucleic Acids Research*, Volume 43, Issue 3, 18 February 2015, Pages 1626–1636, https://doi.org/10.1093/nar/gku1368

The Editors were first alerted in early 2020 that the bands in Figure 4C show unusual levels of similarity. The journal and institution investigated the matter at the time and did not find conclusive evidence to support the allegations.

When similar allegations were brought to the Editors’ attention again in late 2021, the journal re-opened the investigation. With newer techniques, the journal also identified potential concerns with Figure 2D. While the corresponding author works with the institution where the research was conducted to retrieve the original data, the Editors advise readers to examine the details of this study with particular care.

Julian E. Sale, Barry L. Stoddard

Senior Executive Editors

